# The impact of cocaine on diagnosis stability in psychosis, based on a case report

**DOI:** 10.1192/j.eurpsy.2023.1386

**Published:** 2023-07-19

**Authors:** M. Pérez Machado, L. Cano Roch, E. Mur Mila

**Affiliations:** 1 psychiatry, Institute of Neuropsychiatry and addictions; 2 psychiatry, Hospital del Mar, Parc de Salut Mar; 3 psychiatry, Mental Health Research Group, Hospital Del Mar(IMIM), BARCELONA, Spain

## Abstract

**Introduction:**

Substance-induced psychosis (SIP) is the name given to a psychosis that starts in the context of substance abuse, but persists for days and weeks with no substance use. There is growing recognition that individuals with substance-induced psychosis are more likely to develop a schizophrenia spectrum disorder. Early onset of substance-induced psychosis and cannabis use are predictors of conversion. Nevertheless, more evidence is needed to identify other factors.

**Objectives:**

The objective of this study was to analyze the progression of substance-induced psychosis to several mental disorders, by reporting a case of a cocaine user, and identifying the factors that promote the progression.

**Methods:**

We report the case of a 55 years old male, with long-term consumption of endovenous cocaine and heroin, who has experienced various episodes of substance-induced psychosis in the past.

In 2017, he presented haptic and visual hallucinations oriented as parasite delusion during rehab hospitalization. The symptoms disappear after a few days of risperidone treatment and absence of consumption. Consuming cocaine and heroin ev in previous days.In October 2018 and July 2021 the patient was hospitalized in Dual Pathology for similar episodes oriented as substance-induced psychosis.

In the current episode, the patient was hospitalized in the Dual Pathology Unit due to a psychotic episode described as parasite infestation delusion and prejudice delusion against his family. The last consumption of heroin and cocaine was 3 months ago.

**Results:**

**Table 1:** 

** DATE OF HOSPITALIZATION**	** LAST CONSUME BEFORE HOSPITALIZATION**	**HABITUAL COMPSUPTION BEFORE HOSPITALIZATION**	** INICIAL SYMTOMS DURING** **HOSPITALIZATION**	**TREATMENT DURING HOSPITALIZATION**
04/12/17 - 19/12/17 -	1 DAY	COCAINE AND HEROIN: 1/8g/24h ev	-PARASITE DELUSION -Haptic Hallucinations	Risperidone 2mg/day
25/09/18 08/10/18	1 DAY	-COCAINE AND HEROINE: O,5g, 2-3 times a week,ev	-PARASITE DELUSION - Haptic and Visual Hallucinations	Risperidona hasta 4mg/day
15/06/21 - 28/06/21	?	-COCAINE AND HEROINE: O,5g, 2-3 times a week,ev	-PARASITE DELUSION - Behavioral impact	Risperidone 3mg/day
15/09/2022- 17/10/2022	3 MONTHS	-	- PARASITE DELUSION -Behavioural impact - Prejudice delusion	Paliperidone 3mg/day

**Conclusions:**

This case report exemplifies the temporal relationship between substance use and the development of psychotic illness. Suggesting substance-induced psychosis as an indicator for the future development of a severe mental disorder. For this reason, more evidence is needed to identify other factors that promote the progression to severe mental disorders and stablish a higher risk group

**Disclosure of Interest:**

None Declared

